# Sleep disturbances in older surgical patients with and without suspected cognitive impairment: A multicenter cohort study

**DOI:** 10.1371/journal.pone.0318866

**Published:** 2025-02-20

**Authors:** Nina Butris, Ellene Yan, Yasmin Alhamdah, Paras Kapoor, Leif Erik Lovblom, Aparna Saripella, David Gold, Jean Wong, David F. Tang-Wai, Linda Mah, Mark I. Boulos, David He, Frances Chung

**Affiliations:** 1 Department of Anesthesia and Pain Management, Toronto Western Hospital, University Health Network, University of Toronto, Toronto, Ontario, Canada; 2 Institute of Medical Science, Temerty Faculty of Medicine, University of Toronto, Toronto, Ontario, Canada; 3 Biostatistics Department, University Health Network, Toronto, Ontario, Canada; 4 Neuropsychology Clinic, Krembil Neuroscience Centre, Toronto Western Hospital, University Health Network, Toronto, Ontario, Canada; 5 Department of Psychiatry, University of Toronto, Toronto, Ontario, Canada; 6 Division of Neurology, Department of Medicine, University of Toronto, Toronto, Ontario, Canada; 7 Division of Geriatric Psychiatry, Department of Psychiatry, Temerty Faculty of Medicine, University of Toronto, Toronto, Ontario, Canada; 8 Hurvitz Brain Sciences Research Program, Sunnybrook Health Sciences Centre, Toronto, Ontario, Canada; 9 Department of Anesthesiology and Pain Medicine, Mount Sinai Hospital, University Health Network, University of Toronto, Toronto, Ontario, Canada; University of Rijeka Faculty of Medicine: Sveuciliste u Rijeci Medicinski fakultet, CROATIA

## Abstract

**Aims:**

Early detection and management of sleep disturbances can improve postoperative outcomes given the high prevalence of sleep disturbances and unrecognized cognitive impairment in older surgical patients. There is an association between sleep disturbances and cognitive impairment in the general population. However, the relationship in older surgical patients has not been systematically investigated. The objective of this study was to assess the prevalence and trajectory of preoperative and postoperative sleep disturbances in older surgical participants with and without suspected cognitive impairment (sCI).

**Methods:**

Two hundred and fifty-two participants aged ≥ 65 years undergoing non-cardiac surgery were recruited. The primary outcome was the prevalence and trajectory of sleep disturbances measured by the Pittsburgh Sleep Quality Index (PSQI) in participants with and without sCI preoperatively, 30, 90, and 180 days postoperatively. The main exposure, preoperative sCI, was operationalized as screening positive on one or more of the following cognitive screening tools: Centers for Disease Control and Prevention cognitive question (answered “yes”), Ascertain Dementia Eight-item Questionnaire (≥2), Telephone Montreal Cognitive Assessment (≤18), and Modified Telephone Interview for Cognitive Status (≤31). Sleep disturbances were defined as a PSQI score > 5. Mixed effects logistic regression models with random intercepts were used for the dichotomous outcome of sleep disturbances.

**Results:**

One hundred and eight participants (43%) screened positive for preoperative sCI. The prevalence of preoperative sleep disturbances was higher in participants with sCI versus without (63% vs 47%, *P* = 0.02). Postoperatively in both groups, the prevalence of sleep disturbances was lower at 30, 90, and 180 days, compared to the preoperative assessment and overall trajectories did not differ significantly. Female sex and depression were associated with poorer postoperative sleep, regardless of cognitive status.

**Conclusion:**

Sleep disturbances and suspected cognitive impairment are highly prevalent in surgical cohorts. Targeting conditions such as depression which affect sleep, may improve postoperative outcomes.

## Introduction

Sleep disturbances may manifest as abnormal sleep events, such as inadequate sleep duration and sleep disorders. The prevalence of sleep disturbances in a community-dwelling population, as measured by the Pittsburgh Sleep Quality Index (PSQI), ranges between 27–32% [1,2]. In the general population, sleep disturbances compromise the immune system, increase levels of inflammation, and heighten the risk of developing co-morbidities such as hypertension and diabetes [3]. Sleep disturbances can also increase the risk of developing mental health disorders and impair cognitive health [3].

Cognitive impairment (CI) describes deficits in complex attention, executive function, language, learning and memory, perceptual-motor function, and social cognition [4]. The severity range of CI encompasses subjective cognitive decline that does not impede everyday function, mild cognitive impairment (MCI), in which cognitive deficits are present but do not impede everyday function, and dementia, in which several cognitive domains are impaired affecting everyday function [5]. Those with MCI experience more disrupted sleep patterns than healthy individuals [6]. Sleep disturbances are independently associated with a higher risk of developing CI in older community-dwelling participants [7]. Sleep disorders, such as obstructive sleep apnea, also increase the risk of developing CI [8].

Worldwide, older adults contribute to almost 50% of surgeries that occur and are at an increased risk of developing CI [9]. In elective and emergency surgeries, the prevalence of unrecognized CI is 37% and 50%, respectively [10]. However, cognition is not universally assessed in the preoperative setting, and CI may be underappreciated. Preoperative sleep disturbances are common with a prevalence of up to 60% [11]. Both CI and sleep disturbances in older surgical patients have been associated with an increased risk of postoperative complications such as delirium, increased length of hospital stay (LOS), delayed functional recovery, and mortality [12–14].

The early detection and management of sleep disturbances can optimize postoperative recovery, given the high prevalence of sleep disturbances and unrecognized CI in older surgical patients. Despite the association between sleep disturbances and CI in the general population [7], the relationship between sleep disturbances and CI in older surgical patients has not been systematically investigated. The objective of this study was to assess the prevalence and trajectory of sleep disturbances in older surgical participants with and without suspected CI (sCI) preoperatively and 1 to 3, 30, 90, and 180 days postoperatively. We also aimed to assess the preoperative risk factors associated with postoperative sleep disturbances. We hypothesized that participants who screened positive for sCI preoperatively would have a higher prevalence of sleep disturbances and worsening in sleep patterns compared to those who screened negative for sCI at the preoperative assessment and 1 to 3, 30, 90, and 180 days postoperatively.

## Materials and methods

This study was a multicenter (Toronto Western Hospital and Mount Sinai Hospital, Toronto, Ontario, Canada) prospective observational cohort sub-study based on the Post-operative Functional Disability in Unrecognized Cognitive Impairment study. This study involved 252 participants undergoing non-cardiac surgery from January 27^th^, 2022 to December 5^th^, 2023. After research ethics board approval (REB #: 20-6186, and 21-0123-E), participants meeting the inclusion criteria described below were identified consecutively by the research team from preoperative clinics one to 30 days before surgery, and written informed consent was obtained.

### Primary objectives

The objective of this study was to assess the prevalence and trajectory of sleep disturbances in older surgical participants with and without sCI during preoperative assessment and at 1 to 3, 30, 90, and 180 days postoperatively. We also aimed to assess the preoperative risk factors associated with postoperative sleep disturbances.

### Study population

Study participants were recruited from preoperative clinics. The inclusion criteria were (1) age ≥ 65 years old undergoing elective, non-cardiac, primary surgical procedures (i.e., revisions of previous surgical procedures were not included) with an expected LOS of at least two days and (2) available by telephone contact for follow-up. The exclusion criteria were: (1) prior dementia diagnosis, (2) outpatient surgery, (3) clinically evident previous stroke, (4) uncorrected visual and/or hearing impairment, (5) insufficient English fluency to comprehend questionnaires, (6) current alcohol or substance misuse, or (7) inability to comprehend study instructions or provide informed consent.

### Data collection

Preoperative demographic and medical data were obtained from participants and medical records. Sleep disturbances, the primary outcome, were assessed using the Pittsburgh Sleep Quality Index (PSQI) [15] and Single-Item Sleep Quality Scale (SQS) [16] via an online survey (REDCap, an online platform to complete secure surveys: www.project-redcap.org) preoperatively and at 1 to 3, 30, 90, and 180 days postoperatively.

### Pittsburgh Sleep Quality Index (PSQI)

The Pittsburgh Sleep Quality Index (PSQI) is a subjective questionnaire consisting of 19 items assessing sleep over a one-month time interval with an average administration time of 10 minutes [15]. The PSQI contains seven “component” scores with a zero to three Likert scale: subjective sleep quality, sleep latency, sleep duration, habitual sleep efficiency, sleep disturbances, use of sleeping medication, and daytime dysfunction [15]. The global PQSI score is calculated by adding all seven component scores together. The PSQI score ranges from 0 to 21. A global PSQI score > 5 indicates the presence of sleep disturbance, with 89.6% sensitivity and 86.5% specificity [15].

### Single-Item Sleep Quality Scale (SQS)

The Single-Item Sleep Quality Scale (SQS) is a subjective measurement that assesses sleep quality over a seven-day recall period using a visual analogue scale (VAS) (with 0, terrible, to 10, excellent) [16]. When rating sleep quality, participants are asked to consider the following: how many hours of sleep they got, how easily they fell asleep, how often they woke up during the night (except to go to the bathroom), how often they woke up earlier than they needed to in the morning, and how refreshing their sleep was [16].

### Cognitive impairment (CI)

The development of the study protocol and the collection of research data occurred during the COVID-19 pandemic, when preoperative assessment by anesthesiologists was primarily conducted virtually. This necessitated the use of telephone and online assessment tools for CI instead of face-to-face screening tools such as the Mini-Mental State Examination or Montreal Cognitive Assessment. Since there is no single universally accepted screening tool for detecting CI in surgical patients [17] and CI was assessed by phone or online, we chose four screening tools that are easily implemented in preoperative assessment [18]. The Centers for Disease Control and Prevention (CDC) cognitive question [19] and Ascertain Dementia Eight-item Questionnaire (AD8) [20] were used to screen for CI via an online survey. The Telephone Montreal Cognitive Assessment (T-MoCA) [21] which adjusts for education levels, and Modified Telephone Interview for Cognitive Status (TICS-M) [22] were used to assess cognition over the phone. Preoperative sCI referred to screening positive on at least one of the four cognitive screening tools: CDC cognitive question (answered “yes”) [19], AD8 (cutoff ≥ 2) [20], T-MoCA (cutoff ≤ 18) [21] or TICS-M (cutoff ≤ 31) [22].

Each participant was emailed a personalized URL link associated with their participant ID. Participants who were unable to complete the online assessments or did not have regular internet access could opt to complete the assessments via telephone.

### Secondary outcome measurements

The STOP (Snoring, Tiredness, Observed apnea, and High blood pressure) questionnaire was used to assess the risk of obstructive sleep apnea (OSA) [23]. VAS pain for pain [24,25], World Health Organization Disability Assessment Schedule 2.0 (WHODAS 2.0) for functional disability [26,27], Lawton Brody Instrumental Activities of Daily Living Scale for IADL (IADL) [28], FRAIL questionnaire for frailty [29], EuroQol 5 Dimension (EQ-5D-5L) Questionnaire for quality of health [30], and the Geriatric Depression Scale (GDS) (≥5 cut-off for depression) [31] were all administered online at the preoperative assessment, and 30, 90, and 180 days postoperatively.

On postoperative days 1 to 3 or until discharge, sleep disturbances and pain were measured by the SQS and VAS, respectively [16,24,25]. Postoperative delirium was assessed by the short form of the confusion assessment method (short CAM) and chart-based method of delirium [32]. CAM was conducted within 24 hours of admission and re-administered every 12 hours by nursing and research personnel blinded to the information in medical charts.

At 30, 90, and 180 days postoperatively, the same online and telephone assessments as the preoperative assessment were repeated. Postoperative complications, non-home discharge, all-cause mortality, hospital re-admissions, and emergency room visits were also evaluated at 30, 90, and 180 days postoperatively. Three versions of T-MoCA were administered at each postoperative time point to minimize practice effects. One week prior to the scheduled follow-up, participants were notified via email and/or phone to complete the assessments. Additional reminders were sent via email and/or phone call in the event of no response. If participants withdrew, their data collected up to that point were included in the final analysis.

### Statistical analyses

Statistical analyses were performed using R software (Version 4.1.1). Demographic data and assessment outcomes were characterized using descriptive statistics at the preoperative assessment and each follow-up time point. Categorical data were presented as counts and percentages, and continuous data were presented as means and standard deviations (SD) or standard errors (SE) or medians [interquartile range (IQR)]. Parametric continuous data between groups were compared using the Student’s t-test, while non-parametric continuous data were compared using the Mann-Whitney U test. Categorical data were analyzed using Fisher’s exact test and chi-square test. The trajectories of sleep disturbances were compared between participants with and without sCI using linear and logistic mixed-effects regression models.

For the binary outcome of sleep disturbances indicated by a PSQI score > 5, a logistic regression model was used with time, cognition status, time-by-group interaction, and a random participant-level intercept. The unadjusted prevalence of sleep disturbances and model-based comparisons were reported. For time points where the logistic regression models did not converge, we performed a simplified Fisher’s exact test to compare the prevalence of sleep disturbances between participants with and without sCI. Models that also included a random effect for time did not show improvement in the model fit, as assessed using a likelihood ratio test. Model assumptions were assessed by examining residuals. Sensitivity analyses were performed to compare sleep disturbances in participants with and without sCI based on screening positive on each individual CI screening tool. As a second set of sensitivity analyses, the same model structure was used under a linear mixed-effects model for the continuous outcome of PSQI score. Post-hoc pairwise comparisons of the time points were performed using the same model structure. The Tukey- Kramer correction was used to control type I error for assessing associations at multiple time points.

Preoperative factors that might contribute to sleep disturbances at 30, 90, and 180 days postoperatively were assessed using an expanded linear-mixed effects model for the continuous outcome of PSQI scores. The evaluated preoperative factors were cognition, sex, age, pre-existing diseases (the presence of hypertension, diabetes, chronic obstructive pulmonary disease (COPD), or coronary artery disease (CAD)), obstructive sleep apnea (OSA), years of education, STOP questionnaire score, smoking status, surgery type, pain, functional disability, IADL, frailty, and depressive symptoms. Each preoperative factor was added to the existing linear mixed-effects model in a stepwise fashion (the preoperative value was repeated for each of the four time points). We refer to this collection of models as minimally-adjusted models. Multivariable modelling was then performed with stepwise linear regression using factors from the minimally adjusted models with a *P*-value of ≤ 0.2. We also assessed for multicollinearity. Functional disability by WHODAS and quality of life by EQ-5D-5L exhibited a high correlation. Therefore, EQ-5D-5L was not included in the analysis. All the statistical tests were two-tailed, and we used an alpha level of 0.05.

### Sample size estimation

The sample size was calculated using a mixed-effects model analysis approach via the Repeated Measures and Sample Size (RMASS) program (www.rmass.org/). Based on data from a systematic review and meta-analysis, the proportion of participants with preoperative sCI was 46.0% [10]. Using a correlation estimate of 0.5 between preoperative and follow-up PSQI data at 180 days postoperatively and a decrease in PSQI score of 0.006 per day, a sample size of 184 participants will provide 90% power to detect a change in the sCI group compared to the No-sCI group. To account for a 30% dropout rate, the final sample size required would be 239 participants. We set a two-sided alpha value of 0.05 for statistical significance.

## Results

### Study population

**Fig 1:**
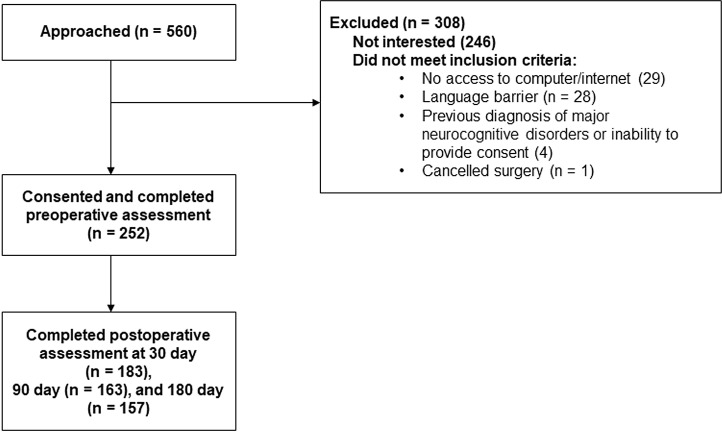
Study flowchart.

A total of 560 participants were approached, and 252 consented and completed the preoperative assessment (Fig 1). Of the 252 participants who completed the preoperative assessment, 42.9% screened positive for preoperative sCI on at least one or more of the four cognitive screening tools which is consistent with a previous systematic review and meta-analysis [10]. The demographic and characteristic data are summarized in Table 1. The median age was 72 years [68–77] and 56% were female. The main type of surgery was orthopedic (65.5%). Fifty percent of participants underwent regional anesthesia. Compared to participants without sCI, participants with sCI were significantly older, had higher body mass index, coronary artery diseases, hypertension and asthma/chronic obstructive pulmonary diseases, and had lower years of education. The demographic and characteristic data of those screened positive for sCI by the individual screening tools are summarized in Supplementary (S)1A, 1B, 1C, and 1D Tables in S1 File.

**Table 1:** Demographic and characteristic data in participants screened positive for CI.

	Preop No-sCI (n = 144)	Preop sCI (n = 108)	P-value
Sex, female	85 (59)	56 (51.9)	0.26
Age (years)	71 [76–68]	73 [77–69]	**0.04**
Education (years)	16 [14–18]	15 [12–16]	**0.008**
Ethnicity/Race			
White	116 (80.6)	84 (77.8)	0.29
Asian	3 (2.1)	4 (3.7)	0.18
Hispanic	5 (3.5)	2 (1.9)	0.70
Other^a^	13 (9)	8 (7.4)	0.99
BMI, kg/m^2^	28.9 ± 6.8	31.8 ± 6.6	**0.002**
ASA Class	2.9 ± 5.6	2.9 ± 5.5	0.19
I	2 (1.4)	0 (0)	
II	27 (18.8)	11 (10.2)	
III	106 (73.6)	87 (80.6)	
IV	9 (6.3)	10 (9.3)	
Comorbidities			
Hypertension	63 (43.8)	70 (64.8)	**0.001**
CAD	5 (3.5)	4 (3.7)	**0.001**
Stroke	3 (2.1)	0 (0)	0.18
Asthma/COPD	23 (16)	7 (6.5)	**0.02**
Smoker	6 (4.2)	0 (0)	0.99
Diabetes mellitus	15 (10.4)	8 (7.4)	0.41
OSA	18 (12.5)	14 (13)	0.91
Restless Leg Syndrome	0 (0)	1 (0.9)	0.43
STOP questionnaire, ≧2	32 (22.2)	23 (21.3)	0.86
Surgical procedure			
Orthopedic	89 (61.8)	76 (70.4)	0.16
General	17 (11.8)	7 (6.4)	0.15
Urological	11 (7.6)	9 (8.3)	0.84
Spinal	7 (4.9)	2 (1.9)	0.31
Otolaryngological	5 (3.5)	6 (5.6)	0.42
Gynecological	6 (4.2)	3 (2.7)	0.74
Other^b^	9 (6.3)	5 (4.6)	0.58
Anesthetics			
RA	65 (45.1)	62 (57.4)	0.05
GA	54 (37.5)	36 (33.3)	0.49
RA + GA	24 (16.7)	10 (9.3)	0.09
Preoperative Medication			
Anticholinergic drugs	1 (0.7)	2 (1.9)	0.58
Sedative hypnotics	8 (5.6)	13 (12)	0.65
Length of stay (days)	1 [1–3]	2 [1–3]	0.48

Values were presented as mean ± standard deviation, median [interquartile range], or number of participants (%) where appropriate.

Abbreviations: ASA: American Society of Anesthesiologists; BMI: body mass index; CAD: coronary artery disease; COPD: chronic obstructive pulmonary disease; CPAP: continuous positive airway pressure; GA: general anesthesia; OSA: obstructive sleep apnea; RA: regional anesthesia; STOP: Snoring, Tiredness, Observed apnea, and High blood pressure); sCI: suspected cognitive impairment.

^a^Other ethnicity/race included Mediterranean, Black, Middle Eastern, Indigenous, and mixed.

^b^Other surgical procedures included abdominal, bariatric, and thoracic procedures.

### Preoperative sleep disturbances

**Fig 2:**
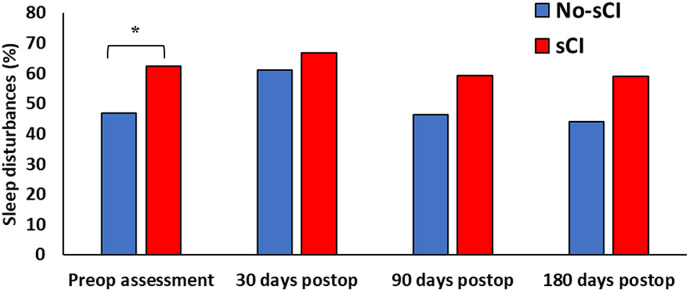
The unadjusted prevalence of sleep disturbances preoperatively and postoperatively at 30, 90, and 180 days postoperatively in sCI vs No-sCI participants. Sleep disturbances was defined as Pittsburgh Sleep Quality Index > 5. sCI; screened positive on at least one of the four cognitive tests. Variables were presented as the number of participants (n, %). * *P* < 0.05 in participants with sCI vs without. Model-based comparisons were reported.

A global PSQI score > 5 defined participants as having sleep disturbances [15]. Both sCI and No-sCI groups had high prevalence of preoperative sleep disturbances, but the prevalence was higher in participants with sCI versus participants without sCI (62.9% vs 46.5%, *P* = 0.02) (Fig 2). Sensitivity analyses demonstrated that preoperative sleep disturbances were higher in participants with sCI compared to participants without sCI using the CDC cognitive question (84% vs 50.2%, *P* = 0.001, logistic regression model did not converge) or the AD8 alone (77.3% vs 48.6%, *P* = 0.0008) (S1 Fig in S1 File). There was no difference in the prevalence of preoperative sleep disturbances between the sCI vs No-sCI groups using the T-MoCA (59.7% vs 50.9%) or TICS-m (50% vs 53.8%) (S1 Fig in S1 File).

### Changes in the prevalence of postoperative sleep disturbances over time

Despite the prevalence of preoperative sleep disturbances being significantly higher in participants with sCI vs. participants without sCI, the between-group difference in prevalence was not significantly higher at 30, 90, and 180 days postoperatively (Fig 2). The No-sCI group had a significantly higher within-group difference in prevalence of sleep disturbances at 30 days postoperatively compared to their preoperative assessment (61.1% vs. 46.5% *P* = 0.008). There was no average difference in the effect of time on the prevalence of sleep disturbance between the two groups, suggesting a comparable rate of improvement across all time points regardless of cognitive status (*P* for interaction = 0.7). Sensitivity analyses demonstrated that at 30 days postoperatively, the prevalence of sleep disturbances was higher in participants with sCI vs participants without sCI using the CDC cognitive question (100% vs 59.2%, *P* = 0.0003) or the AD8 (77.8% vs 60.6%, *P* = 0.03) (S1 Fig in S1 File). At 90 and 180 days postoperatively, there were no significant differences in sleep disturbances when using CDC or AD8 alone (S1 Fig in S1 File). There were no differences in the prevalence of sleep disturbances at 30, 90, and 180 days postoperatively in the sCI nor No-sCI group using the T-MoCA or TICS-m (S1 Fig in S1 File).

Of the 252 participants, 165 (65.5%) underwent orthopedic surgery, and 53 (21%) had general, urological, or gynecological surgery. The prevalence of postoperative sleep disturbances at 30 and 180 days in participants undergoing orthopedic surgery versus other types of surgeries was significantly higher (80.7% vs. 31.6%, *P* = < .0001; 58% vs. 23.5%, *P* = 0.03) (S2 Fig in S1 File).

### Changes in scores of PSQI postoperatively over time

**Fig 3:**
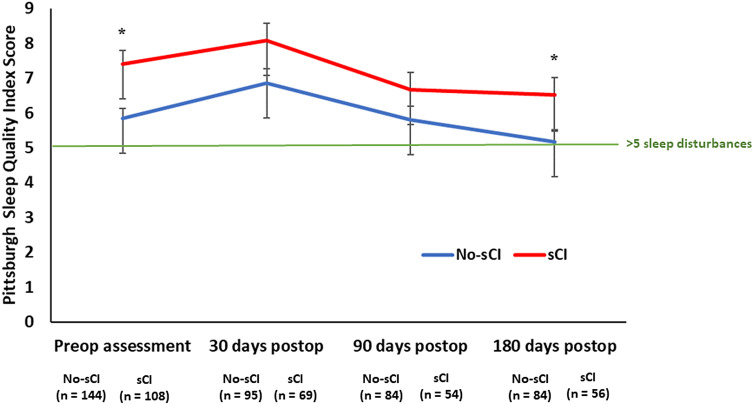
Mean Pittsburgh Sleep Quality Index (PSQI) scores at preoperative assessment, 30, 90, and 180 days postoperatively in sCI vs No-sCI participants. The PSQI is scored from 0 to 21. The green line indicates the mean PSQI score of 5. A score greater than 5 indicates sleep disturbances. sCI (suspected cognitive impairment): screened positive on at least one of the four cognitive tests. Error bars represent standard error. * *P* < 0.05 in participants with sCI vs participants without.

When sleep disturbances were examined as a continuous variable, the preoperative and 180 days postoperative PSQI scores were significantly higher in the sCI than the No-sCI group (7.4 ± 0.4 vs. 5.8 ± 0.8, *P* = 0.001; 6.5 ± 0.5 vs. 5.2 ± 0.4, *P* = 0.03) (Fig 3).

The sCI group had a significantly higher PSQI score 30 days postoperatively (8.1 ± 0.5) compared to their scores at 90 days postoperatively (6.7 ± 0.5, *P* = 0.009) and 180 days postoperatively (6.5 ± 0.5, *P* = 0.001). The No-sCI group had a significantly higher PSQI score 30 days postoperatively (6.8 ± 0.4) compared to their scores at preoperative assessment (5.8 ± 0.3, *P* = 0.0005), 90 days postoperatively (5.8 ± 0.4, *P* = 0.004), and 180 days postoperatively (5.2 ± 0.4, *P* < .0001).

There was no average difference in the effect of time on PSQI scores in both groups (*P* for interaction = 0.6). Sensitivity analyses demonstrated that using the CDC, PSQI scores were higher in participants with sCI vs participants without sCI preoperatively, 30, 90, and 180 days postoperatively (S2 Table; S3 Fig in S1 File). Using the AD8, PSQI scores were higher in participants with sCI vs participants without sCI preoperatively, 30, and 180 days postoperatively (S2 Table; S3 Fig in S1 File). There were no significant differences in preoperative and postoperative PSQI scores in participants with sCI vs participants without sCI using the T-MoCA or TICS-m (S2 Table; S3 Fig in S1 File).

### Patient-centered outcome measures

The sCI group with preoperative sleep disturbances had significantly greater pain levels, functional disability, frailty, and depressive symptoms, and lower quality of health than the sCI group without preoperative sleep disturbances at preoperative assessment (Table 2).

**Table 2:** Assessment of sCI participants with and without preoperative sleep disturbances.

Assessment	No Preop Sleep Disturbances (n = 40)	Preop Sleep Disturbances (n = 68)	P-value
VAS pain	4.5 ± 0.5	5.5 ± 0.4	**0.04**
WHODAS 2.0	7.9 ± 1.2	12.2 ± 0.9	**0.004**
IADL	7.2 ± 0.2	6.6 ± 0.2	0.05
FRAIL	1.2 ± 0.2	1.7 ± 0.1	**0.02**
EQ-5D-5L	0.7 ± 0.03	0.6 ± 0.03	**0.001**
GDS	1.9 ± 0.5	4.6 ± 0.4	**<.0001**

All values are presented as mean ± standard error. Sleep disturbance: Pittsburgh Sleep Quality Index (PSQI) > 5. Abbreviations: EQ-5D-5L: European Quality of Life 5 Dimensions 3 Level Version; FRAIL: Fatigue, Resistance, Ambulation, Illnesses, and Loss of weight; GDS: Geriatric Depression Scale; IADL: Independent Activities of Daily Living; Preop: Preoperative; sCI: suspected cognitive impairment; VAS pain: Visual Analogue Scale.

### Postoperative days 1 to 3

Of the 252 participants, 154 completed the postoperative day 1 assessment, 69 completed the day 2 assessment, and 40 completed the day 3 assessment. Reasons for not completing the assessments included early discharge, ICU admission, or refusal. There were no significant differences in sleep quality as measured by the SQS or pain levels as measured by VAS pain between groups on postoperative days 1 to 3 (*P* > 0.05) (S3 Table).

Six participants (2%) screened positive for postoperative delirium from chart-based method of delirium and/or CAM during their hospital stay. Of the six participants with delirium, five had both preoperative CI and sleep disturbances. Between sCI and No-sCI participants, there were no significant differences in postoperative complications, non-home discharge, all-cause mortality, hospital re-admissions, and emergency room visits.

### Preoperative factors associated with sleep disturbances over time

Linear mixed-effects models were used to assess the preoperative factors associated with postoperative sleep disturbance scores. Importantly, we found that female sex and depressive symptoms were associated with poorer sleep across all time points (Table 3).

**Table 3:** Minimally and fully adjusted multivariable analyses of preoperative factors significantly associated with sleep disturbances at 30-, 90-, and 180-day postoperatively.

*Cognitive Impairment detected using any one of the four * *CI* * tools*					
Covariate	Comparison	Minimally Adjusted Model		Fully Adjusted Model	
		Estimate (95% CI)	*P*-value	Estimate (95% CI)	*P*-value
Cognition	sCI *vs* No-sCI	1.6 (0.7, 2.4)	0.0004	0.54 (−2.89, 1.38)	0.2
**Sex**	**Male** *vs* **Female**	−**1.43 (**−**2.12,** −**0.73)**	**<0.001**	−**1.05 (**−**1.70,** −**0.4)**	**0.0017**
Age	+1	−0.007 (−0.07, 0.06)	0.83	–	–
Pre-existing diseases	Yes *vs* no	0.54 (−0.28, 1.36)	0.2	0.21 (−0.53, 0.95)	0.58
OSA	Yes *vs* no	0.28 (−0.65, 1.2)	0.56	–	–
Years of education	+1	−0.03 (−0.15, 0.1)	0.67	–	–
STOP questionnaire	+1	0.41 (0.07, 0.75)	0.02	0.19 (−0.12, 0.5)	0.24
Smoker	Yes *vs* no	−0.99 (−2.71, 0.73)	0.26	–	–
Surgery type	Orthopedic vs. other	1.36 (0.63, 2.1)	0.003	0.44 (−0.28, 1.17)	0.23
VAS pain	+1	0.33 (0.21, 0.45)	<0.001	0.12 (−0.02, 0.26)	0.09
WHODAS	+1	0.17 (0.12, 0.23)	<0.001	0.05 (−0.04, 0.13)	0.32
IADL	+1	−0.48 (−0.77, −0.2)	0.001	−0.09 (−0.4, 0.23)	0.59
FRAIL	+1	1 (0.69, 1.33)	<0.001	0.33 (−0.07, 0.73)	0.11
**GDS**	+**1**	**3.1 (2.1, 4.1)**	**<0.001**	**3.71 (0.91, 2.97)**	**0.0003**

Sleep disturbances are defined as Pittsburgh Sleep Quality Index > 5. Estimates were from linear mixed effects models.

Abbreviations: AD8: Ascertain Dementia Eight-item Questionnaire; CDC: Centers for Disease Control and Prevention; FRAIL: Fatigue, Resistance, Ambulation, Illnesses, and Loss of weight; GDS: Geriatric Depression Scale; IADL: Instrumental Activities of Daily Living; OSA: Obstructive Sleep Apnea; Preop: Preoperative; sCI: suspected cognitive impairment; STOP: Snoring, Tiredness, Observed apnea, high blood Pressure; TICS-m: Modified Telephone Interview for Cognitive Status; T-MoCA: Telephone Montreal Cognitive Assessment; VAS: Visual Analogue Scale; WHODAS, World Health Organization Disability Assessment Schedule.

The evaluated preoperative factors were cognition, sex, age, pre-existing diseases (the presence of hypertension, diabetes, chronic obstructive pulmonary disease, or coronary artery disease), OSA, years of education, STOP questionnaire score, smoking status, surgery type, pain, disability, IADL, frailty, and depressive symptoms. *P* ≤ 0.2 in minimally adjusted analysis and *P* ≤ 0.05 in fully adjusted analysis were accepted as statistically significant.

## Discussion

Worldwide, there is an increasing number of older adults who are having surgeries yearly and who are at an increased risk of developing postoperative complications such as delirium and mortality [9,12–14]. Older adults are also at an increased risk of developing CI, which was shown to be associated with an increased risk of postoperative complications [13]. Our recent systematic review and meta-analysis indicated that preoperative sleep disturbances are common with a high prevalence of 60% but there is a lack of literature on sleep disturbances in older surgical patients with cognitive impairment [11]. In the current study, we found that the prevalence of preoperative sleep disturbances was higher in patients who screened positive on at least one of the four screening tools for sCI than No-sCI groups by 16.4%. Similarly, a higher prevalence of sleep disturbances was found in those screening positive on the CDC cognitive question or AD8, but not T-MoCA or TICS-m, using sensitivity analyses.

In the postoperative period, sleep disturbances decreased over time at 30, 90, and 180 days in both sCI and No-sCI groups without differences in the trajectories, suggesting that sCI does not modify the effect of time on sleep disturbances. The decrease in sleep disturbances in both sCI and No-sCI groups may be attributed to the reduction in anxiety, pain, and fear related to surgery and anesthesia as patients recover [33]. We also showed that participants who were female or had greater depressive symptoms were more likely to have poorer postoperative sleep scores across all time points.

Comparing the sCI group without preoperative sleep disturbances, our novel findings were that the sCI group with preoperative sleep disturbance had significantly greater pain levels, functional disability, frailty, and depressive symptoms, and lower quality of health at preoperative assessment. More attention needs to focus on the perioperative care of patients with both sCI and sleep disturbances, as they tend to experience postoperative sleep disturbances due to factors such as noise, light, and monitoring in the hospital environment [12]. Sleep quality can be improved by providing patients with non-pharmacological interventions such as ear plugs or eye masks, reducing light, implementing protocols that minimize disturbance, avoiding multi-person rooms, or if necessary, administrating pharmacological treatments such as zolpidem, dexmedetomidine, and melatonin [12].

Importantly, we found a substantial number of surgical participants who screened positive for sCI. Interestingly, we observed differences in sleep disturbances preoperatively and 30 days postoperatively between sCI and No-sCI groups using the CDC cognitive question or AD8, but not with the T-MoCA or TICS-m. We postulated that there may be a decrease in the heterogeneity of CI within groups with CDC cognitive question or AD8. The CDC cognitive question asks a single question regarding subjective cognitive complaints [19]. The AD8 is more suited for screening dementia [20], whereas the MoCA is better for screening for MCI [21]. Thus, the AD8 can identify participants with more severe CI and had greater sleep disturbances [34].

There are several proposed mechanisms to explain the association between CI and a higher overall prevalence of sleep disturbances. Poor sleep quality can lead to neuroinflammation which can disrupt neurogenesis in hypothalamic regions responsible for learning and memory [35]. CI can cause structural and functional changes to the brain affecting regions responsible for sleep regulation such as the hypothalamus, thalamus, and brain nuclei [36]. Sleep disturbances can also disrupt gamma-aminobutyric acid and cyclic adenosine monophosphate neuronal pathways, affecting memory consolidation and normal brain functioning [36]. Acetylcholine is also responsible for regulating sleep and memory consolidation. However, its levels decrease with aging, leading to the decline of cholinergic cells in the basal forebrain [37]. The glymphatic system consists of metabolic waste product removal such as amyloid-beta proteins (involved in Alzheimer’s disease), in the cerebral spinal fluid (CSF) flow through the brain [37]. Sleep disturbances can disrupt the glymphatic system, impairing the removal of metabolic waste products and increasing the risk of developing CI [38]. Impaired sleep is associated with increased levels of CSF proteins such as Tau proteins, which are involved with CI [38].

When comparing demographic characteristics, our study demonstrated participants with sCI had more hypertension cases compared to participants without sCI. This suggests the sCI group may be more likely to have vascular disease, vascular cognitive impairment or ischemic white matter changes which may alter sleep [39].

Our study elucidated some of the characteristics associated with preoperative sleep disturbance and CI. We showed that sCI group with preoperative sleep disturbances had a higher level of pain than the sCI group without sleep disturbances. Sleep and pain have a well-established bidirectional relationship where pain leads to sleep disturbances and poor sleep further exacerbates pain [40]. While the relationship between sleep and pain is well-established, the underlying mechanisms for this association are not fully yet understood. One hypothesized mechanism is changes in dopamine signaling caused by sleep disturbances which affect pain processes [41]. Additionally, sleep disturbances increase the levels of pro-inflammatory markers such as interleukin-6 and C-reactive protein which have a significant association with pain [41]. Addressing sleep disturbances and managing pain could optimize cognitive function in older surgical patients and reduce the risk of developing postoperative complications such as delirium, discharge to assisted care, readmission, and mortality [13].

Another novel finding was that participants with sCI and preoperative sleep disturbances had greater preoperative depressive symptoms than participants with sCI and without sleep disturbances. Participants with and without sCI who had greater symptoms of preoperative depression had poorer sleep at 30, 90, and 180 days postoperatively. In our study, the incidence of preoperative severe depression was 2.4% which is similar to the 3.4% incidence in a community setting [42]. Preoperative sleep disturbances in the sCI group may be associated not only with sCI itself, but also with psychological factors such as depression, which is known to be linked to cognitive impairment and can further exacerbate sleep disturbances [43,44].

Poor sleep can lead to the development of depression or the worsening of symptoms through reductions in prefrontal cortex activity, impaired immune system function, and increased inflammation [45]. A meta-analysis demonstrated that those with sleep disorders, such as insomnia, had a higher risk of major depressive disorder than those without [46]. Improving depressive symptoms through cognitive-behavioral therapy, anti-depressants, and other treatments can improve sleep quality in patients with depression and insomnia [47,48].

It is important to highlight certain limitations of this study. Firstly, sCI was assessed using remote screening tools and not by physician diagnosis nor a comprehensive evaluation of cognition. To date, no rapid cognitive screening tools have been validated in a surgical population [17]. Due to the COVID-19 pandemic, virtual preoperative assessments were done, which necessitated the use of telephone and online assessment tools. With no single universally accepted screen for detecting CI, we operationalized sCI as screening positive on at least one of the four cognitive screening tools. This may have overestimated the prevalence of CI. Nevertheless, we provided sensitivity analysis on each of the four screening tools which provided similar results in those screened positive for the CDC cognitive question or the AD8. Although participants with a prior dementia diagnosis were excluded, individuals with mild cognitive impairment and dementia that had not been previously diagnosed were likely included. However, we cannot ascertain which individuals specifically met the criteria for MCI or dementia based solely on the screening tools used.

Secondly, confounding bias from CI may have impacted subjective awareness or perception of sleep quality. Thirdly, there may be a self-selection bias as limited language capability and cultural differences may be limiting factors for cognitive screening. A high level of education was observed in our participant sample, and findings may not be generalizable to those with lower education levels. Another limitation is sleep disturbances were measured by a questionnaire and participants were not formally tested with objective measurements. There may be additional factors contributing to sleep disturbances that may not be appreciated by a participant but could be determined via polysomnography. The preoperative assessments included tools such as the WHODAS-2.0, rely on a one-month recall period which may have captured factors unrelated to surgery during the preoperative assessment. Lastly, the sample size remains relatively small although it was consistent with the calculated power of the study. This may limit the generalizability of the findings to larger populations.

## Conclusions

The prevalence of sleep disturbances in the sCI group was sixteen percent higher than the No-sCI group at the preoperative assessment. Sleep patterns improved in both groups over time postoperatively. Female sex and depressive symptoms, regardless of cognitive status, were associated with poor postoperative sleep across all time points. Identifying sleep disturbance and CI in surgical cohorts and targeting treatable conditions like depressive symptoms may enhance surgical outcomes.

## Supporting information

S1 File This file contains supplementary figures and tables referenced in the manuscript. (DOCX)
