# Supplementary material for: Sleep disturbances in older surgical patients with and without suspected cognitive impairment: A multicenter cohort study
Source: PLoS One. 2025 Feb 20;20(2):e0318866. doi: 10.1371/journal.pone.0318866 (PMC11841874; doi:10.1371/journal.pone.0318866)
Supplement: S1 File — (DOCX) [file pone.0318866.s001.docx]

**S1A Table. Demographic data in participants with and without suspected cognitive impairment (sCI) using the Centers for Disease Control and Prevention (CDC) cognitive question.**

|  | Preop No-sCI  (n = 227) | Preop sCI  (n = 25) | P-value |
| --- | --- | --- | --- |
| Sex, female | 130 (57.3) | 11 (44) | 0.2 |
| Age (years) | 72 [68–77] | 74 [71–77] | 0.1 |
| Education (years) | 16 [13–16] | 15 [12–18] | 0.2 |
| Ethnicity/Race |  |  |  |
| White | 181 (79.7) | 19 (76) | 0.2 |
| Asian | 6 (2.6) | 1 (4) | 0.7 |
| Hispanic | 6 (2.6) | 1 (4) | 0.7 |
| Other^a^ | 17 (7.5) | 4 (16) | 0.1 |
| BMI, kg/m^2^ | 29.9 ± 7 | 31 ± 5.8 | 0.2 |
| ASA Class | 2.9 ± 0.5 | 3.1 ± 0.4 | 0.3 |
| I | 2 (0.9) | 0 (0) |  |
| II | 37 (16.3) | 1 (4) |  |
| III | 172 (75.8) | 21 (84) |  |
| IV | 16 (7) | 3 (12) |  |
| Comorbidities |  |  |  |
| Hypertension | 114 (50.2) | 19 (76) | **0.01** |
| CAD | 8 (3.5) | 1 (4) | 0.9 |
| Stroke | 3 (1.3) | 0 (0) | **0.007** |
| Asthma/COPD | 29 (12.8) | 1 (4) | 0.2 |
| Smoker | 6 (2.6) | 0 (0) | 0.9 |
| Diabetes mellitus | 19 (8.4) | 4 (16) | 0.2 |
| OSA | 25 (11) | 7 (28) | **0.02** |
| STOP questionnaire, ≧2 | 48 (21.1) | 7 (28) | 0.4 |
| Surgical procedure |  |  |  |
| Orthopedic | 150 (66.1) | 15 (60) | 0.5 |
| General | 22 (9.7) | 2 (8) | 0.8 |
| Urological | 16 (7) | 4 (16) | 0.1 |
| Spinal | 8 (3.5) | 1 (4) | 0.9 |
| Otolaryngological | 7 (3.1) | 4 (16) | **0.003** |
| Gynecological | 8 (3.5) | 1 (4) | 0.9 |
| Other^b^ | 11 (4.8) | 3 (12) | 0.2 |
| Anesthetics |  |  |  |
| RA | 107 (47.1) | 21 (84) | **<0.001** |
| GA | 72 (31.7) | 18 (72) | **<0.001** |
| RA + GA | 31 (13.7) | 3 (23) | 0.8 |

Values were presented as mean ± standard deviation, median [interquartile range], or number of participants (%) where appropriate. Abbreviations: ASA: American Society of Anesthesiologists; BMI: body mass index; CAD: coronary artery disease; COPD: Chronic obstructive pulmonary disease; CPAP: continuous positive airway pressure; GA: general anesthesia; OSA: obstructive sleep apnea; RA: regional anesthesia; STOP: Snoring, Tiredness, Observed apnea, and High blood pressure); CI: suspected cognitive impairment. ^a^Other ethnicity/race included Mediterranean, Black, Middle Eastern, Indigenous, and mixed. ^b^Other surgical procedures included abdominal, bariatric, and thoracic procedures.

**S1B Table. Demographic data in participants with and without suspected cognitive impairment (sCI) using Ascertain Dementia Eight-item Questionnaire (AD8).**

|  | Preop No-sCI  (n = 208) | Preop sCI  (n = 44) | P-value |
| --- | --- | --- | --- |
| Sex, female | 115 (55.2) | 26 (59) | 0.4 |
| Age (years) | 72 [68–77] | 72.5 [69–77] | 0.08 |
| Education (years) | **16 [13.5**–**16]** | **14 [12**–**16]** | **0.004** |
| Ethnicity/Race |  |  |  |
| White | 165 (79.3) | 35 (79.5) | 0.9 |
| Asian | 6 (2.9) | 1 (2.3) | 0.8 |
| Hispanic | 5 (2.4) | 2 (4.5) | 0.4 |
| Other^a^ | 15 (7.2) | 6 (13.6) | 0.2 |
| BMI, kg/m^2^ | 29.5 ± 7.3 | 32.1 ± 6.6 | **0.01** |
| ASA Class | 2.8 ± 0.5 | 3.2 ± 0.5 | 0.07 |
| I | 2 (1) | 0 (0) |  |
| II | 36 (17.3) | 2 (4.5) |  |
| III | 157 (75.5) | 36 (81.8) |  |
| IV |  |  |  |
| Comorbidities |  |  |  |
| Hypertension | 103 (49.5) | 30 (68.2) | **0.02** |
| CAD | 8 (3.8) | 1 (2.3) | 0.6 |
| Stroke | 3 (1.4) | 0 (0) | 0.2 |
| Asthma/COPD | 30 (14.4) | 0 (0) | 0.3 |
| Smoker | 6 (2.9) | 0 (0) | 0.9 |
| Diabetes mellitus | 19 (9.1) | 4 (9.1) | 0.9 |
| OSA | 15 (7.2) | 17 (38.6) | **< .00001** |
| STOP questionnaire, ≧2 | 33 (15.9) | 22 (50) | **< .00001** |
| Surgical procedure |  |  |  |
| Orthopedic | 135 (64.9) | 30 (68.1) | 0.7 |
| General | 22 (10.6) | 2 (4.5) | 0.2 |
| Urological | 15 (7.2) | 5 (11.4) | 0.4 |
| Spinal | 9 (4.3) | 0 (0) | 0.7 |
| Otolaryngological | 8 (3.8) | 3 (6.8) | 0.4 |
| Gynecological | 7 (3.4) | 2 (4.5) | 0.3 |
| Other^b^ | 13 (6.3) | 1 (2.3) | 0.3 |
| Anesthetics |  |  |  |
| RA | 101 (48.6) | 26 (59) | 0.2 |
| GA | 76 (36.5) | 14 (31.8) | 0.6 |
| RA + GA | 30 (14.4) | 4 (9.1) | 0.3 |

Values were presented as mean ± standard deviation, median [interquartile range], or number of participants (%) where appropriate. Abbreviations: ASA: American Society of Anesthesiologists; BMI: body mass index; CAD: coronary artery disease; COPD: Chronic obstructive pulmonary disease; CPAP: continuous positive airway pressure; GA: general anesthesia; OSA: obstructive sleep apnea; RA: regional anesthesia; STOP: Snoring, Tiredness, Observed apnea, and High blood pressure); sCI: suspected cognitive impairment. ^a^Other ethnicity/race included Mediterranean, Black, Middle Eastern, Indigenous, and mixed. ^b^Other surgical procedures included abdominal, bariatric, and thoracic procedures.

**S1C Table. Demographic data in participants with and without suspected cognitive impairment (sCI) using the Telephone Montreal Cognitive Assessment (T-MoCA).**

|  | Preop No-sCI  (n = 175) | Preop sCI  (n = 77) | P-value |
| --- | --- | --- | --- |
| Sex, female | 104 (59.4) | 37 (48) | 0.09 |
| Age (years) | 73 [69–77] | 72 [68–76] | 0.08 |
| Education (years) | 16 [14–18] | 15 [12–16] | **0.001** |
| Ethnicity/Race |  |  |  |
| White | 142 (81.1) | 58 (75.3) | 0.3 |
| Asian | 3 (1.7) | 4 (5.2) | 0.1 |
| Hispanic | 5 (2.9) | 2 (2.6) | 0.9 |
| Other^a^ | 14 (8) | 7 (9.1) | 0.7 |
| BMI, kg/m^2^ | 29.5 ± 7.1 | 31.2 ± 6.3 | **0.03** |
| ASA Class | 2.9 ± 0.5 | 3 ± 0.5 | 0.9 |
| I | 2 (1.1) | 0 (0) |  |
| II | 28 (16) | 10 (13) |  |
| III | 133 (76) | 60 (77.9) |  |
| IV | 11 (6.3) | 8 (10.4) |  |
| Comorbidities |  |  |  |
| Hypertension | 85 (48.6) | 48 (62.3) | **0.04** |
| CAD | 4 (2.3) | 5 (6.5) | 0.1 |
| Stroke | 3 (1.7) | 0 (0) | 0.5 |
| Asthma/COPD | 20 (11.4) | 10 (13) | 0.7 |
| Smoker | 5 (2.9) | 1 (1.3) | 0.9 |
| Diabetes mellitus | 10 (5.7) | 3 (3.9) | 0.5 |
| OSA | 20 (11.4) | 12 (15.6) | 0.4 |
| STOP questionnaire, ≧2 | 40 (22.9) | 15 (19.5) | 0.5 |
| Surgical procedure |  |  |  |
| Orthopedic | 110 (62.9) | 55 (71.4) | 0.2 |
| General | 19 (10.9) | 5 (6.5) | 0.3 |
| Urological | 15 (8.6) | 5 (6.5) | 0.6 |
| Spinal | 7 (4) | 2 (2.6) | 0.6 |
| Otolaryngological | 6 (3.4) | 5 (6.5) | 0.3 |
| Gynecological | 8 (4.6) | 1 (1.3) | 0.2 |
| Other^b^ | 10 (5.7) | 4 (5.2) | 0.9 |
| Anesthetics |  |  |  |
| RA | 82 (46.9) | 45 (58.4) | 0.09 |
| GA | 67 (38.2) | 23 (29.9) | 0.2 |
| RA + GA | 25 (14.3) | 9 (11.7) | 0.6 |

Values were presented as mean ± standard deviation, median [interquartile range], or number of participants (%) where appropriate. Abbreviations: ASA: American Society of Anesthesiologists; BMI: body mass index; CAD: coronary artery disease; COPD: Chronic obstructive pulmonary disease; CPAP: continuous positive airway pressure; GA: general anesthesia; OSA: obstructive sleep apnea; RA: regional anesthesia; STOP: Snoring, Tiredness, Observed apnea, and High blood pressure); sCI: suspected cognitive impairment. ^a^Other ethnicity/race included Mediterranean, Black, Middle Eastern, Indigenous, and mixed. ^b^Other surgical procedures included abdominal, bariatric, and thoracic procedures.

**S1D Table. Demographic data in participants with and without suspected cognitive impairment (sCI) using the Telephone Interview for Cognitive Status (TICS-m).**

|  | Preop No-sCI  (n = 234) | Preop sCI  (n = 18) | P-value |
| --- | --- | --- | --- |
| Sex, female | 134 (57.3) | 7 (38.9) | 0.1 |
| Age (years) | 72 [68–77] | 75 [69.5–78] | 0.08 |
| Education (years) | 16 [13–16] | 13 [12–15.8] | **0.04** |
| Ethnicity/Race |  |  |  |
| White | 189 (80.8) | 11 (61.1) | 0.05 |
| Asian | 6 (2.6) | 1 (5.6) | 0.5 |
| Hispanic | 7 (3) | 0 (0) | 0.2 |
| Other^a^ | 17 (7.3) | 4 (22.2) | 0.03 |
| BMI, kg/m^2^ | 29.9 ± 7 | 31.9 ± 5.2 | 0.1 |
| ASA Class | 2.9 ± 0.5 | 3.1 ± 0.4 | 0.6 |
| I | 2 (0.9) | 0 (0) |  |
| II | 37 (15.8) | 1 (5.6) |  |
| III | 178 (76) | 15 (83.3) |  |
| IV | 17 (7.3) | 2 (11.1) |  |
| Comorbidities |  |  |  |
| Hypertension | 123 (52.6) | 10 (55.6) | 0.8 |
| CAD | 8 (3.4) | 1 (5.6) | 0.6 |
| Stroke | 2 (0.9) | 1 (5.6) | 0.08 |
| Asthma/COPD | 27 (11.6) | 3 (16.7) | 0.5 |
| Smoker | 3 (1.3) | 3 (16.7) | **< .00001** |
| Diabetes mellitus | 19 (8.1) | 4 (22.2) | 0.05 |
| OSA | 27 (11.5) | 5 (27.8) | 0.05 |
| STOP questionnaire, ≧2 | 49 (20.9) | 6 (33.3) | 0.2 |
| Surgical procedure |  |  |  |
| Orthopedic | 151 (64.5) | 14 (77.8) | 0.3 |
| General | 23 (9.8) | 1 (5.6) | 0.6 |
| Urological | 19 (8.1) | 1 (5.6) | 0.7 |
| Spinal | 8 (3.4) | 1 (5.6) | 0.6 |
| Otolaryngological | 11 (4.7) | 0 (0) | 0.8 |
| Gynecological | 9 (3.8) | 0 (0) | 0.4 |
| Other^b^ | 11 (4.7) | 3 (16.7) | **0.03** |
| Anesthetics |  |  |  |
| RA | 116 (49.6) | 11 (61.1) | 0.3 |
| GA | 85 (36.3) | 5 (27.8) | 0.5 |
| RA + GA | 32 (13.7) | 2 (11.1) | 0.8 |

Values were presented as mean ± standard deviation, median [interquartile range], or number of participants (%) where appropriate. Abbreviations: ASA: American Society of Anesthesiologists; BMI: body mass index; CAD: coronary artery disease; COPD: Chronic obstructive pulmonary disease; CPAP: continuous positive airway pressure; GA: general anesthesia; OSA: obstructive sleep apnea; RA: regional anesthesia; STOP: Snoring, Tiredness, Observed apnea, and High blood pressure); sCI: suspected cognitive impairment. ^a^Other ethnicity/race included Mediterranean, Black, Middle Eastern, Indigenous, and mixed. ^b^Other surgical procedures included abdominal, bariatric, and thoracic procedures.

**S2 Table. Mean Pittsburgh Sleep Quality Index (PSQI) Scores preoperatively and postoperatively at 30, 90, and 180 days in participants with and without suspected cognitive impairment (sCI) (individual screening tool).**

| ***Any one of the four cognitive screening tools*** | | | | | | | | |
| --- | --- | --- | --- | --- | --- | --- | --- | --- |
| **Assessment** | **Preoperatively** | | **30 days postoperatively** | | **90 days postoperatively** | | **180 days postoperatively** | |
|  | **No-sCI** n = 144 | **sCI** n = 108 | **No-sCI** n = 95 | **sCI** n = 69 | **No-sCI** n = 84 | **sCI** n= 54 | **No-sCI** n = 84 | **sCI** n = 56 |
| **PSQI Score, Mean** | 5.8 ± 0.3 | **7.4 ± 0.4*** | 6.8 ± 0.4 | 8.1 ± 0.5 | 5.8 ± 0.4 | 6.7 ± 0.5 | 5.2 ± 0.4 | **6.5 ± 0.5*** |

| ***CDC cognitive question*** | | | | | | | | |  |
| --- | --- | --- | --- | --- | --- | --- | --- | --- | --- |
| **Assessment** | **Preoperatively** | | **30 days postoperatively** | | **90 days postoperatively** | | **180 days postoperatively** | |  |
|  | **No-sCI**  n = 227 | **sCI** n = 25 | **No-sCI** n = 147 | **sCI** n = 17 | **No-sCI** n = 124 | **sCI** n= 14 | **No-sCI** n = 126 | **sCI** n = 14 |  |
| **PSQI Score, Mean** | 6.2 ± 0.2 | **9.4 ± 0.7**** | 7.4 ± 0.3 | **9.9 ± 0.8*** | 6.1 ± 0.3 | **8.0 ± 0.9*** | 5.5 ± 0.3 | **8.7 ± 0.9**** |  |
| ***AD8*** | | | | | | | | | |
|  | | **No-sCI** n = 208 | **sCI** n = 44 | **No-sCI** n = 137 | **sCI** n = 27 | **No-sCI** n = 115 | **sCI** n = 23 | **No-sCI** n = 115 | **sCI** n = 25 |
| **PSQI Score** | | 6.1 ± 0.3 | **8.5 ± 0.6**** | 7.2 ± 0.3 | **9.4 ± 0.7*** | 6.1 ± 0.3 | 7.3 ± 0.7 | 5.5 ± 0.3 | **7.5 ± 0.7**** |
| ***T-MoCA*** | | | | | | | | | |
|  | | **No-sCI** n = 175 | **sCI** n = 77 | **No-sCI** n = 114 | **sCI** n = 50 | **No-sCI** n = 101 | **sCI** n = 37 | **No-sCI** N = 106 | **sCI**  N = 34 |
| **PSQI Score** | | 6.3 ± 0.3 | 7.1 ± 0.4 | 7.4 ± 0.3 | 7.9 ± 0.5 | 6.1 ± 0.3 | 6.6 ± 0.5 | 5.7 ± 0.3 | 6.4 ± 0.5 |
| ***TICS-m*** | | | | | | | | | |
|  | | **No-sCI** n = 234 | **sCI** n = 18 | **No-sCI** n = 154 | **sCI** n = 10 | **No-sCI** n = 130 | **sCI** n = 8 | **No-sCI**  n = 134 | **sCI** n = 6 |
| **PSQI Score** | | 6.5 ± 0.2 | 6.8 ± 0.9 | 7.6 ± 0.3 | 6.9 ± 1.1 | 6.3 ± 0.3 | 6.1 ± 1.2 | 5.9 ± 0.3 | 5.3 ± 1.3 |

Abbreviations: AD8: Ascertain Dementia Eight-item Questionnaire; CDC: Centers for Disease Control and Prevention; sCI: suspected cognitive impairment; TICS-m: Telephone Interview for Cognitive Status; T-MoCA: Telephone Montreal Cognitive Assessment. The PSQI is scored from 0 to 21. A score greater than 5 indicates sleep disturbances. * P<0.05, **P<0.001 in No-sCI vs. sCI participants. All values are presented as mean ± SE.

**S3 Table. Assessment in participants with and without suspected cognitive impairment (sCI) on postoperative day 1 to day 3 (n = 154).**

|  | POD 1 | | POD 2 | | POD 3 | |
| --- | --- | --- | --- | --- | --- | --- |
|  | **No-sCI  (n = 84)** | **sCI (n = 70)** | **No-sCI  (n = 34)** | **sCI  (n = 35)** | **No-sCI  (n = 22)** | **sCI  (n = 18)** |
| SQS | 4.3 ± 0.3 | 3.9 ± 0.3 | 6 ± 0.5 | 5.5 ± 0.5 | 5.7 ± 0.6 | 5.4 ± 0.7 |
| VAS pain | 4.4 ± 0.3 | 4 ± 0.3 | 4.2 ± 0.4 | 4.6 ± 0.5 | 3.8 ± 0.5 | 3.8 ± 0.6 |
| Delirium,  n (%) | 0 (0) | 2 (2.9) | 0 (0.0) | 2 (5.7) | 1 (4.5) | 1 (5.6) |

All values are presented as mean ± SE unless otherwise stated. Abbreviations: POD: Postoperative day; sCI: suspected cognitive impairment; SQS: Single-Item Sleep Quality Scale; VAS pain: Visual Analogue Scale. * P<0.05 in No-sCI vs. sCI participants.

**S4 Table. Minimally adjusted and fully adjusted multivariable analyses of preoperative factors significantly associated with postoperative sleep disturbances at 30, 90, and 180 days postoperatively in both participants with and without suspected cognitive impairment (sCI) (individual screening tool).**

| *Cognitive Impairment detected using CDC cognitive question* | | | | | |
| --- | --- | --- | --- | --- | --- |
| Covariate | **Comparison** | **Minimally Adjusted Model** | | **Fully Adjusted Model** | |
|  |  | **Estimate (95% CI)** | ***P*-value** | **Estimate (95% CI)** | ***P*-value** |
| Cognition | **sCI *vs* No-sCI** | **3.16 (1.75, 4.57)** | **<0.001** | **2.0 (0.69, 3.38)** | **0.0032** |
| Sex | **Male *vs* Female** | **-1.48 (-2.17, -0.79)** | **<0.001** | **-1.13 (-1.77, -0.49)** | **0.0006** |
| GDS | **+1** | **2.95 (1.92, 3.97)** | **<0.001** | **1.76 (0.74, 2.78)** | **0.0008** |
| *Cognitive Impairment detected using AD8* | | | | | |
| Covariate | **Comparison** | **Minimally Adjusted Model** | | **Fully Adjusted Model** | |
|  |  | **Estimate (95% CI)** | ***P*-value** | **Estimate (95% CI)** | ***P*-value** |
| Cognition | sCI *vs* No-sCI | 2.4 (1.29, 3.52) | <0.001 | 0.75 (-0.38, 1.88) | 0.1943 |
| Sex | **Male *vs* Female** | **-1.3 (-2, -0.6)** | **0.0003** | **-** **1.04 (-1.68, -0.39)** | **0.0018** |
| GDS | **+1** | **2.94 (1.89, 4)** | **<0.001** | **1.93 (0.9, 3)** | **0.0003** |
| *Cognitive Impairment detected using T-MoCA* | | | | | |
| Covariate | **Comparison** | **Minimally Adjusted Model** | | **Fully Adjusted Model** | |
|  |  | **Estimate (95% CI)** | ***P*-value** | **Estimate (95% CI)** | ***P*-value** |
| Cognition | sCI *vs* No-sCI | 3.16 (1.75, 4.57) | <0.001 | 0.35 (-0.52, 1.21) | 0.43 |
| Sex | **Male *vs* Female** | **-1.41 (-2.13, -0.69)** | **0.0001** | **-1.06 (-1.71, -0.41)** | **0.0015** |
| GDS | **+1** | **3.36 (2.36, 4.37)** | **<0.001** | **2 (0.98, 3)** | **0.0001** |
| *Cognitive Impairment detected using TICS-m* | | | | | |
| Covariate | **Comparison** | **Minimally Adjusted Model** | | **Fully Adjusted Model** | |
|  |  | **Estimate (95% CI)** | ***P*-value** | **Estimate (95% CI)** | ***P*-value** |
| Cognition | sCI *vs* No-sCI | 3.16 (1.75, 4.57) | <0.001 | -0.058 (-1.58, 1.47) | 0.94 |
| Sex | **Male *vs* Female** | **-1.3 (-2.06, -0.61)** | **0.0003** | **-1.03 (-1.68, -0.37)** | **0.0023** |
| VAS Pain | **+1** | **0.37 (0.26, 0.49)** | **<0.001** | **0.13 (0.002, 0.26)** | **0.047** |
| GDS | **+1** | **3.36 (2.35, 4.37)** | **<0.001** | **1.99 (0.96, 3.02)** | **0.0002** |

Sleep disturbances are defined as Pittsburgh Sleep Quality Index >5 Estimates were from linear mixed effects models. Abbreviations: AD8: Ascertain Dementia Eight-item Questionnaire; CDC: Centers for Disease Control and Prevention; FRAIL, fatigue, resistance, ambulation, illnesses, and loss of weight; GDS, Geriatric Depression Scale; IADL, instrumental activities of daily living; OSA: Obstructive Sleep Apnea; sCI: suspected cognitive impairment; TICS-m: Modified Telephone Interview for Cognitive Status; T-MoCA: Telephone Montreal Cognitive Assessment; VAS: Visual Analogue Scale; WHODAS, World Health Organization Disability Assessment Schedule. P ≤ 0.2 in univariable analysis and P≤0.05 in multivariable analysis were accepted as statistically significant.

**S1 Figure. Unadjusted prevalence of sleep disturbances preoperatively and postoperatively in suspected cognitive impairment (sCI) (individual screening tool) vs No-sCI participants.**

| 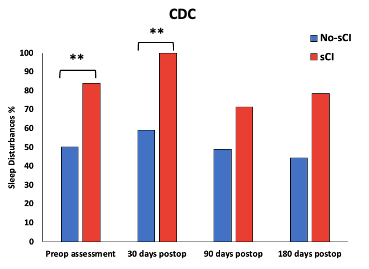 | 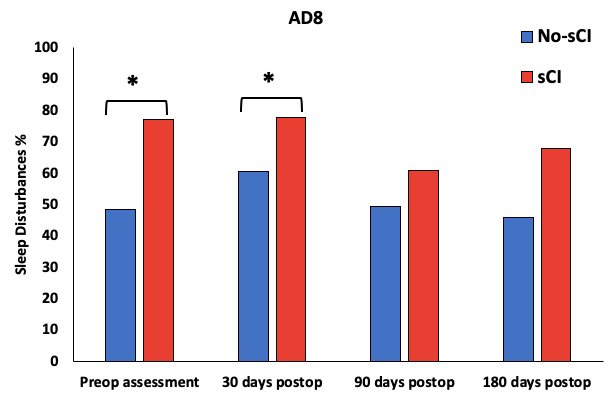 |
| --- | --- |
| 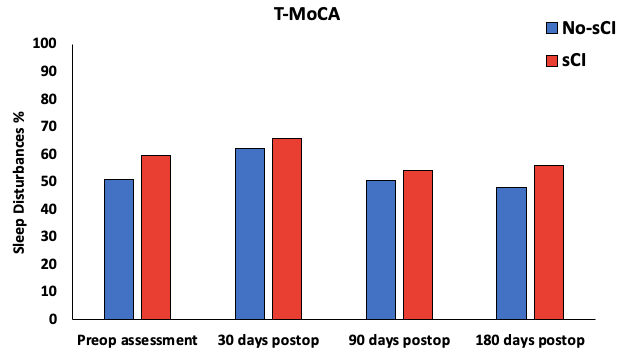 | 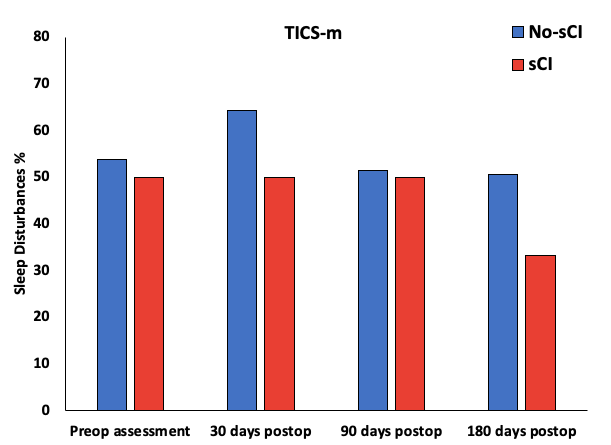 |

Sleep disturbances are defined as Pittsburgh Sleep Quality Index >5. sCI; screened positive on at least one of the four cognitive tests. Variables were presented as the number of participants (n, %). Abbreviations: AD8: Ascertain Dementia Eight-item Questionnaire; CDC: Centers for Disease Control and Prevention; sCI: suspected cognitive impairment; TICS-m: Telephone Interview for Cognitive Status; T-MoCA: Telephone Montreal Cognitive Assessment. * P<0.05, **P<0.001 in sCI vs No-sCI participant.

**S2 Figure. Prevalence of sleep disturbances preoperatively and postoperatively in participants undergoing orthopedic surgery vs. non-orthopedic surgery.**


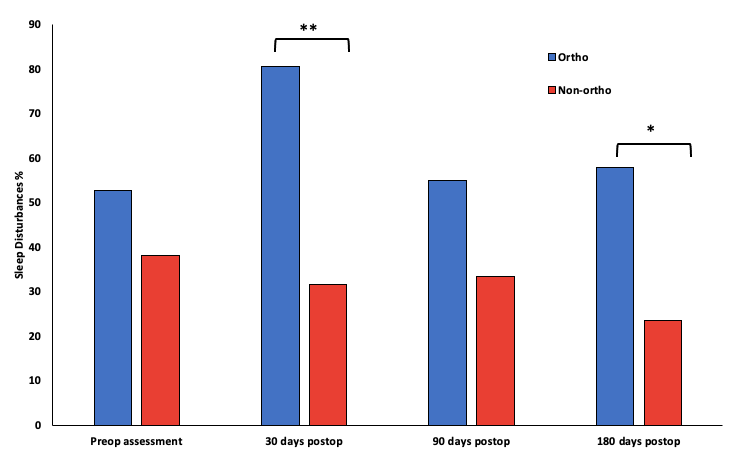


Sleep disturbances are defined as Pittsburgh Sleep Quality Index >5. Variables were presented as the number of participants (n, %). Non-orthopedic surgery included general, urological, spinal, otolaryngological, and gynecological.

**S3 Figure. Mean Pittsburgh Sleep Quality Index (PSQI) scores at preoperative assessment, 30, 90, and 180 days postoperatively in sCI (individual screening tool) vs No-sCI participants.**

| 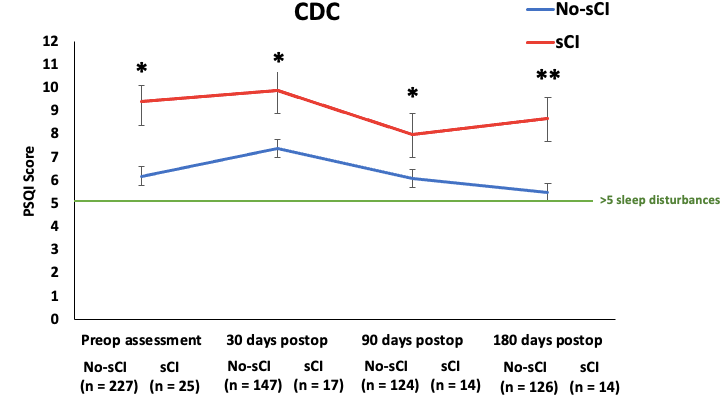 | 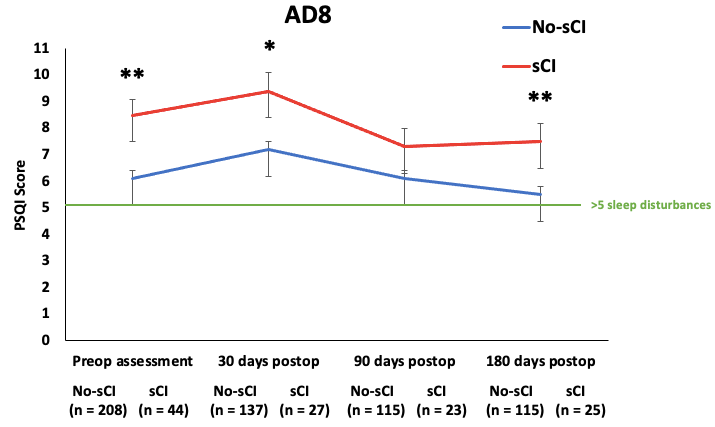 |
| --- | --- |
| 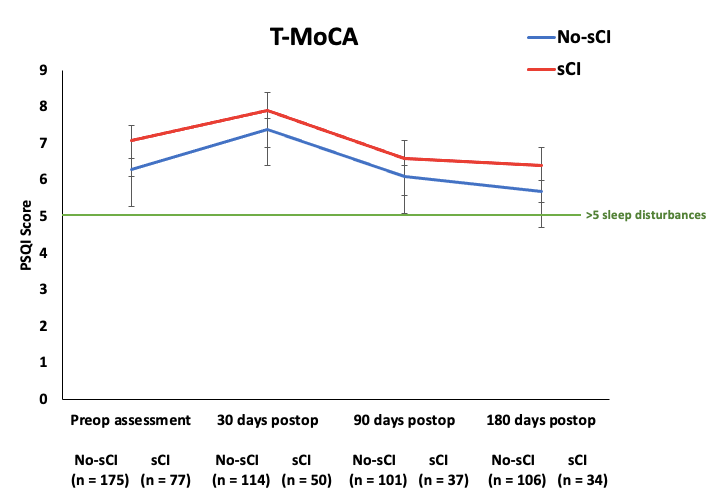 | 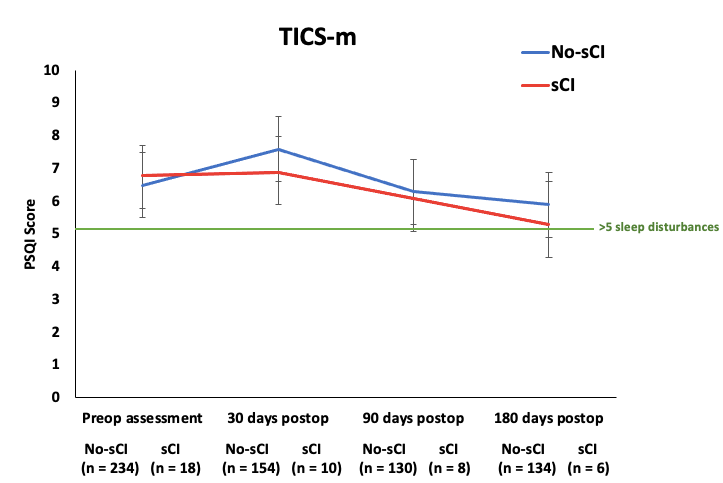 |

|  |
| --- |

The PSQI is scored from 0 to 21. The green line indicates the mean PSQI score of 5. A score greater than 5 indicates sleep disturbances. Error bars represent standard error. Abbreviations: AD8: Ascertain Dementia Eight-item Questionnaire; CDC: Centers for Disease Control and Prevention; sCI: suspected cognitive impairment; TICS-m: Telephone Interview for Cognitive Status; T-MoCA: Telephone Montreal Cognitive Assessment. * P<0.05, **P<0.001 in sCI vs No-sCI participants.
